# Family Meal Environment Differentially Conditions the Prospective Association between Early Childhood Screen Time and Key Social Relationships in Adolescent Girls

**DOI:** 10.3390/children11020145

**Published:** 2024-01-24

**Authors:** Kianoush Harandian, Beatrice Necsa, Tracie A. Barnett, Linda S. Pagani

**Affiliations:** 1 School of Psycho-Education, University of Montreal, Montreal, QC H3C 3J7, Canada; kianoush.harandian@umontreal.ca (K.H.); beatrice.necsa@umontreal.ca (B.N.); 2 School Environment Research Group, University of Montreal, Montreal, QC H3C 3J7, Canada; 3 Sainte-Justine’s Hospital Research Center, Montreal, QC H3T 1C5, Canada; 4 Department of Family Medicine, McGill University, Montreal, QC H3S 1Z1, Canada

**Keywords:** Screen time, family meals, social relationships, early childhood, longitudinal study

## Abstract

*Background:* Despite screen time recommendations, children are increasingly spending time on electronic devices, rendering it an important risk factor for subsequent social and developmental outcomes. Sharing meals could offer a way to promote psychosocial development. This study examines the interaction between family meal environment and early childhood screen time on key adolescent social relationships. *Methods:* Participants are 1455 millennial children (49% boys) from the Quebec Longitudinal Study of Child Development birth cohort. Parents reported on child screen use at ages 2 and 6 years and family meal environment quality at age 6 years. Parents and children reported on parent–child relationships and peer victimization experiences, respectively, at age 13 years. Sex-stratified multiple regression estimated the direct association between screen time trends, family meal environment quality, and their interaction on later social relationship outcomes. *Results:* For girls, when preschool screen time increased, sharing family meals in high-quality environments was associated with more positive and less conflictual relationships with their mothers, whereas meals shared in low- and moderate-quality environments were associated with fewer instances of victimization by their peers. Non-linear associations were not significant for boys. *Conclusion:* Capitalizing on family meal environment represents a simple/cost-efficient activity that can compensate for some long-term risks associated with increased screen use, above and beyond pre-existing and concurrent individual and family characteristics. Public health initiatives may benefit from considering family meals as a complementary intervention strategy to screen use guidelines.

## 1. Family Meal Environment Differentially Conditions the Prospective Association between Early Childhood Screen Time and Key Social Relationships in Adolescent Girls

By its accessibility and versatility, screen use has become a technological epidemic. Children and adolescents are dedicating more time to sedentary screen use, establishing lasting habits of inactivity [1]. Despite the recommendations from the American Academy of Pediatrics (AAP) discouraging more than 1 h of screen media exposure per day for toddlers beyond the age of 2 years, only one-third meet these recommendations [2,3]. Parents often fail to implement proper screen-use restrictions in their homes [4]. On the contrary, screen media is employed as a parenting tool to occupy, calm, and reward children [5].

A growing body of research has repeatedly established screen use in infancy as a risk factor for subsequent executive functioning, academic performance, social competency, as well as emotional and physical health [6,7,8]. Consistently high and increasing screen media trajectories have been suggested to be detrimental to verbal and social interactions in childhood [9,10]. Inadequate development of such skills could impede on one’s ability to interact with others many years later. However, research findings on risks and benefits of screen time are somewhat ambiguous. A narrative review revealed that some types of screen-based activities can show favorable associations with attention, problem solving, and internalizing problems in youth [11]. As such, further examination into the associations between early screen time trends and social outcomes is warranted.

Most research pertaining to screen exposure focuses on the amount of time spent on devices, but less examines the changes in screen use. Studies on trends of screen exposure in early childhood find that between one-quarter and one-half of children show increased use of screens during this critical developmental period [12,13]. These trends are often characterized by different rates of screen time growth [14]. Children with increasing screen time in early childhood showed poorer cognitive and socioemotional development at school entry [13]. Still, little is known about the implications of these early screen time trends on well-being once the school-age years begin. 

The various associations between screen use and well-being are most prominently explained by the Newtonian concept of time displacement. This hypothesis posits that time spent on screens is time that is not dedicated to developmentally appropriate enriching activities and social interactions [15]. By devoting more time to televiewing, children are interacting with family members less and missing out on opportunities to play with peers. This could have repercussions on the family environment as well as their ability to form meaningful relationships [10]. 

On the one hand, it is possible that even with excessive screen use, children could benefit from participating in other stimulating activities that might compensate for the time spent in less enriching activity. However, particularly in early childhood, missed learning opportunities from social interactions might have a significant and lasting impact on social skill development [16]. At this age, the family environment is the main vehicle of socialization, central in achieving key developmental experiences [17]. Hence, offering opportunities to witness and to partake in positive social interactions within the family could be more beneficial to children with greater screen time trends. 

Family meals are daily activities that provide this opportunity for family members to communicate, express feelings, and strengthen relationships [18]. Studies in the United States and in Australia report that roughly two-thirds of preschool-aged children dine with their family at least 5 times a week, rendering it a recurrent family experience [19,20]. Family mealtime habits are relatively constant throughout time, showing some change during transitional periods [21]. Sharing meals as a family may benefit children as they not only foster communication and family connectedness, but also have long-term health benefits such as higher levels of general fitness, healthier eating habits, and better psychosocial adjustment [22,23]. More specifically, mealtime is perceived as a valuable moment to socialize [24]. For children, sharing family meals contributes to feeling loved by parents, perceived emotional support, enjoying family time, and improving parent–child communication [22]. Partaking in this valuable moment also promotes positive social skills and decreases problematic behaviors [25]. This contributes to positive development, which could counter the risks screen time poses on later psychosocial well-being [26,27]. Still, knowledge on the contribution of early family meal sharing on social development is restrained to the few studies on the topic and limited by the use of cross-sectional designs.

Moreover, it is important that research examines not only the long-term repercussions of early screen exposure on development, but further investigates the impacts of screen time trends. The existing body of research also neglects to consider competing explanations of child development with confounders typically limited to sex, socioeconomic status, and parental education. By doing so, observed relationships could be explained by individual or environmental factors that have not typically been accounted for and that could predispose certain individuals to reacting more intensely to some experiences [28]. Furthermore, possible protective experiences that may alleviate the relationship between screen time and later development have yet to be explored. Finally, girls and boys have different experiences in regard to both screen use and family meals [29,30,31]. To this day, girls and boys experience risk and protective factors differently due to biological and contextual factors, emphasizing the need for research designs considering girls and boys as distinct populations [32].

Using the Quebec Longitudinal Study of Child Development (QLSCD) birth cohort, we explore the role of family meal environment on the prospective associations between early screen habits and social relationships in adolescence for girls and boys. In doing so, we investigate the role of both parent-reported screen time trends from ages 2 to 6 years and family meal environment quality at age 6 years on parent- and child-reported key developmental relationships at age 13 years. While controlling for pre-existing and concurrent family and child characteristics, we expect that better family meal environment quality will buffer the associations between early screen time trends and adolescent outcomes. We anticipate distinct associations for girls and boys but offer no directional hypotheses. 

## 2. Methods

### 2.1. Participants

Participants in this study are from the Quebec Longitudinal Study of Child Development (QLSCD), conducted by the Institut de la Statistique du Québec (https://www.jesuisjeserai.stat.gouv.qc.ca/default_an.htm, accessed on 20 December 2023). The QLSCD originates from a stratified sample of 2837 infants randomly selected from the provincial birth registry, between spring 1997 and spring 1998 in Quebec, Canada. After initial selection, 438 families refused to participate, 93 neonates were deemed ineligible, and 186 neonates were untraceable or unreachable. For the first data collection at age 5 months, 2120 infants and their families served as the original sample. At every longitudinal follow-up thereafter, informed consent was obtained annually from parents during early childhood and then biennially from parents and teachers during school-age years. Assent was obtained from children until age 15 years, after which they provided informed consent. This IRB-approved study uses a subsample of 714 boys and 741 girls with complete parent-reported data on screen use at both ages 2 and 6 years who were followed-up at age 6 years for family meal environment quality and again at age 13 years for social relationship outcomes. 

### 2.2. Measures: Predictor. Screen Time Trend (Ages 2 to 6 Years)

At age 2 years, parents reported on the child’s daily time spent (a) watching television programs, (b) watching video cassettes, (c) playing on the computer, and (d) playing video games. At age 6 years, parents once more reported on time spent (a) watching television and (b) playing video games on an average day. A sum of the screen time is calculated for both waves, representing average daily screen time in hours. The trend in screen use refers to the difference between screen times at ages 2 and 6 years. This measure captures the change in screen time, which Pagani et al. have shown to be a strong predictor of subsequent academic, psychosocial, and physical well-being in children from the QLSCD birth cohort [33]. 

### 2.3. Measures: Moderator. Family Meal Environment Quality (Age 6 Years)

An eight-item scale of the family meal environment quality, as reported by parents, inspired by the QLSCD family meal data was used (α = 0.61; mealtimes are enjoyable for everyone; mealtimes are a rush [RC]; mealtimes give us time to talk to each other; and mealtimes include arguments between adults and/or children [RC]; we express feelings to each other; there are lots of bad feelings in our family [RC]; we feel accepted for what we are; and we confide in each other) [31]. To create this scale, we used statements validated in other studies to assess meal enjoyment or atmosphere at family meals, as well as some statements from the McMaster Model of Family Functioning [34,35]. Each item is rated on a Likert scale with options including never or fully disagree (1), occasionally or disagree (2), often enough or agree (3), and always or fully agree (4). A mean of the eight items was calculated. High scores indicate higher family meal environment quality. 

### 2.4. Measures: Outcomes. Key Developmental Social Relationships (Age 13 Years)

#### 2.4.1. Positive Relationship with Parent

Mothers and fathers reported on the relationship with their child (5 items, α_mother_ = 0.82 and α_father_ = 0.80: child talks to you about his/her personal affairs, his/her feelings; you talk to your child about his/her plans for the future (education, career, family, etc.); the time you spend with your child is pleasant; your child talks to you about what he/she does outside of school; and your child talks to you about what happens to him/her at school). Items are inspired by concepts in the Parent-Child Communication Scale [36]. Answers were given on a Likert scale including never (0), seldom (1), sometimes (2), often (3), and very often (4). A mean score of the items was calculated and converted to a scale ranging from 0 to 10 where a higher score indicates a more positive parent–child relationship.

#### 2.4.2. Conflictual Relationship with Parent

The conflictual nature of the parent–child relationship is reported by mothers and fathers (3 items, α_mother_ = 0.62 and α_father_ = 0.67: you punish your child; you argue with your child about school; you argue with your child about his/her friends (acquaintances)). This scale includes factors of punishment and communication, similar to those in Pagani et al. [37]. Items are rated on a Likert scale ranging from never (0) to very often (4). A mean score of the items was calculated and converted to a scale ranging from 0 to 10, where higher scores indicate more conflictual parent–child relationships.

#### 2.4.3. Victimization

Children self-reported on the frequency at which they experienced intimidation from their classmates since the beginning of grade 7 (7 items, α = 0.81: someone called me names, insulted me or said mean things to me; someone didn’t let me be part of his or her group when I wanted to; someone pushed, shoved, hit or kicked me; someone said bad things behind my back to other students; someone made fun of me, laughed at me; I was “taxed” by other students (someone made me pay them or give them something so they would leave me alone); and I was a victim of cyberbullying (insults, threats, intimidation, etc.) on the Internet or by cellphone (perpetrated by other students)). All items, derived from the Revised Class Play [38], were rated on a Likert scale including never (1), once (2), a few times (3), often (4), and very often (5). A mean score was calculated, where higher scores indicate higher frequency peer victimization. 

#### 2.4.4. Measures: Confound Controls (Ages 5 Months to 13 Years)

Child and family characteristics were considered to statistically isolate the screen time predictor from potential pre-existing and concurrent confounds. Individual characteristics include directly measured child body mass index (BMI) at age 1.5 years by a research assistant (0 = below the median, 1 = above the median); child temperament at age 1.5 years, reported by both parents, using items from the National Institute of Mental Health-Diagnostic Interview Schedule (NIMH-DIS; 20 items, α = 0.83; 0 = below the median, 1 = above the median) [39]; neurocognitive skills, measured by a trained examiner, using the Imitation Sorting Task at age 2 years [40]; and self-reported screen time at age 13 years (0 = less than 2 h a day, 1 = more than 2 h a day) [8]. Parent-reported family characteristics at age 5 months include maternal depressive symptoms using a 13-item abridged version of the Center for Epidemiologic Studies Depression Scale (CES-D, α = 0.81; 0 = below the median, 1 = above the median) [41]; parental antisocial antecedents using the NIMH-DIS (12 items, α = 0.61; 0 = below the median, 1 = above the median) [39]; and maternal education (0 = finished high school; 1 = did not finish high school). Parent-reported family characteristics at age 1.5 years include maternal BMI (0 = below the median, 1 = above the median) and family dysfunction (9 items, α = 0.84; 0 = below the median, 1 = above the median) [34]. Parent-reported family characteristics at age 2 years include family configuration (0 = intact, 1 = non-intact); family income (0 = sufficient revenue, 1 = insufficient revenue; as defined by the Canadian low-income cut-off of that year provided by Statistics Canada) [42]; and effective parenting practices using the Parenting Practices Scale (5 items, α = 0.61; 0 = above the median, 1 = below the median) [43].

### 2.5. Data Analytic Procedures

In this study, we examine long-term social risks associated with increases in early childhood screen time as moderated by family meal environment quality. We conducted a series of ordinary least squares multiple regressions (SPSS v.26) in which outcomes at age 13 years were regressed on a continuous estimate of change in total daily screen exposure from ages 2 to 6 years and on family meal environment quality at age 6 years. Moderation analyses then tested the interaction between family meal environment quality on screen time trend and social relationship outcomes using PROCESS 4.0. Analyses were stratified by sex. To obtain an unbiased estimation of the observed effects and limit the possibility of omitted variable bias, pre-existing and concurrent potential child and family confounders were included. 

**Figure 1:**
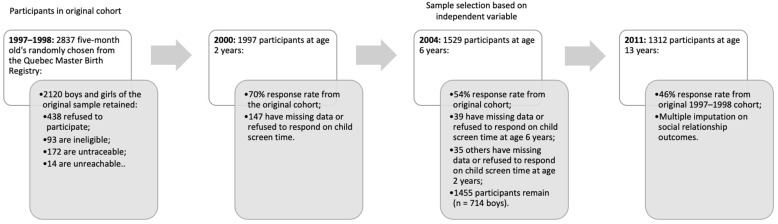
Flow chart displaying the decay from the original sample to the analytical sample.

This study used follow-up data collected from multiple sources at several time points. Figure 1 displays a flow chart of the decay of participants from the original to the study sample. Attrition analyses, presented in the Appendix A, were conducted comparing retained participants with incomplete data to those with complete data (41.9%). We conducted multiple imputation to correct for attrition bias.

## 3. Results

Table 1 reports descriptive statistics for the predictor, moderator, control, and outcome variables. Girls dedicated on average 1.75 h per day at age 2 years and 3.00 h per day at age 6 years to screen use, indicating an additional average 1.25 h of exposure each day. Boys were exposed to a 1.31 h per day increase in screen use from ages 2 to 6 years, with an average exposure of 1.78 h per day and 3.09 h per day at ages 2 and 6 years, respectively. Reported family meal environment quality was high, with mean scores of 3.42 for girls and 3.41 for boys. Positive relationships with both mothers and fathers are left-skewed, whereas conflictual relationships with both parents are right-skewed. Average victimization scores are low, suggesting that most participants do not have remarkably problematic relationships with their parents or their peers.

Table 2 reports unstandardized regression coefficients with standard errors reflecting the adjusted relationship between baseline child/family characteristics between ages 5 months and 13 years and screen time trend from ages 2 to 6 years and family meal environment quality at age 6 years. Notably, for both boys and girls, family dysfunction (β = 0.33, *p* ≤ 0.001, 95% confidence interval [CI], −0.26 to −0.17 for girls and β = 0.28, *p* ≤ 0.001, 95% CI, −0.24 to −0.14 for boys) and less effective parenting practices (β = 0.10, *p* ≤ 0.01, 95% CI, −0.11 to −0.02 for girls and β = 0.17, *p* ≤ 0.001, 95% CI, −0.16 to −0.07 for boys) predicted lower family meal environment quality years later.

**Figure 2:**
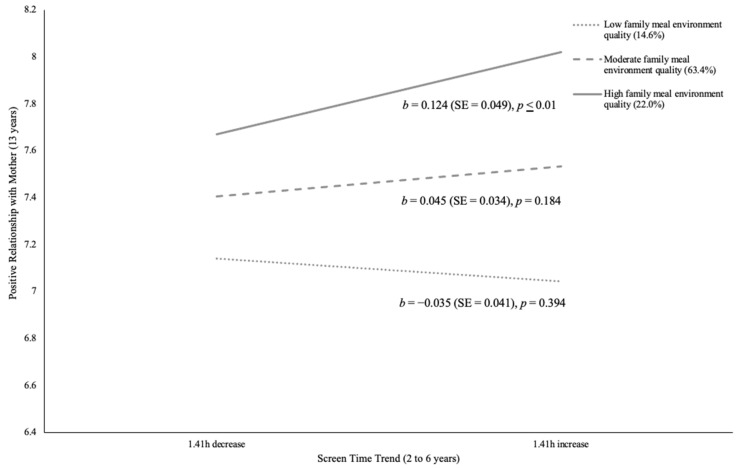
Decomposition of the Adjusted Interaction Between Family Meal Environment Quality at Age 6 Years with Screen Time Trend from Ages 2 to 6 Years Associated with Positive Relationships with Mothers at Age 13 Years for Girls.

**Figure 3:**
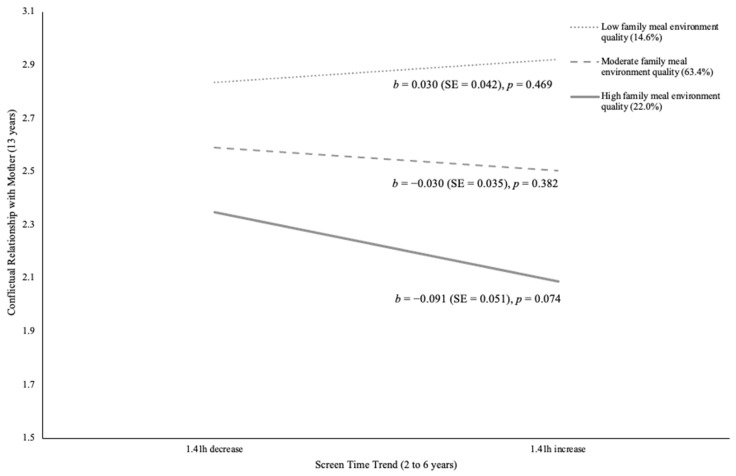
Decomposition of the Adjusted Interaction Between Family Meal Environment Quality at Age 6 Years with Screen Time Trend from Ages 2 to 6 Years Associated with Conflictual Relationships with Mothers at Age 13 Years for Girls.

**Figure 4:**
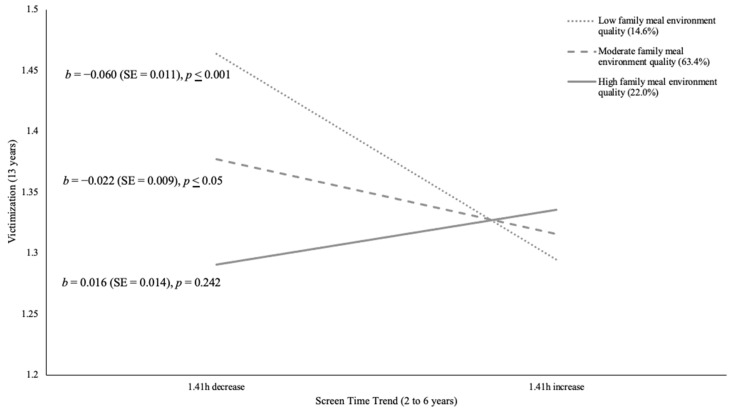
Decomposition of the Adjusted Interaction Between Family Meal Environment Quality at Age 6 Years with Screen Time Trend from Ages 2 to 6 Years Associated with Victimization at Age 13 Years for Girls.

Table 3 documents the unstandardized regression coefficients (standard error) reflecting the adjusted relationship between screen time trends from ages 2 to 6 years and social relationship outcomes at age 13 years, moderated by family meal environment quality at age 6 years for girls. Direct associations between screen time trends as well as family meal environment quality and social relationship outcomes are described in Appendix B. Associations between screen time trends and social relationships were somewhat conditional on family meal environment quality for girls. In fact, mealtime environment amplified the positive association between increased screen time and positive mother–daughter relationships (β = 0.08, *p* ≤ 0.01, 95% CI, 0.06 to 0.43). Specifically, for daughters experiencing high family meal environment quality, higher screen time trends were associated with more positive relationships with mothers. Figure 2 illustrates the decomposition of this adjusted interaction. Conversely, family meal environment quality intensified the negative association between screen time trend and conflictual mother–daughter relationship (β = −0.06, *p* ≤ 0.05, 95% CI, −0.38 to 0.00). Nonetheless, neither high, moderate, nor low quality levels of family meal environment showed significant associations between mother–daughter conflict and screen time trend. Figure 3 shows the decomposition of this adjusted interaction on conflictual mother–daughter relationships. Moreover, girls with low and moderate family meal environment quality revealed a significant negative association between changes in screen time and peer victimization (β = 0.15, *p* ≤ 0.001, 95% CI, 0.07 to 0.17). In other words, these girls experienced less peer victimization when they had a higher early childhood screen time trend. Figure 4 illustrates the decomposition of the adjusted interaction on peer victimization. Girls with higher family meal environment quality were generally less likely to be victimized, though they reported the highest level of victimization when screen time trend increased by 1.41 h.

For boys, family meal environment quality at age 6 years did not moderate the interaction between preschool screen time trend and key social relationship outcomes at age 13 years, as presented in Table 4.

## 4. Discussion

For quite some time, we have known that younger children are increasingly spending time on electronic devices. In fact, many parents believe their children spend too much time in front of screens, yet they continue to employ them as parenting tools to keep their children busy and to reward good behavior [5,44]. This increasing time spent on screens can take away from opportunities to engage in meaningful interactions and developmentally appropriate activities [15]. Nevertheless, screens are now engrained in our daily lives, creating a need for compensatory activities. Sharing family meals can provide opportunities to promote social skills and connect with family, which are often lacking in children with greater screen use [45]. 

In this study, all participants showed better social relationships with parents and peers when partaking in family meals characterized by positive emotions and self-expression. These findings align with previous cross-sectional studies that have shown that family meals are associated with better interactions with parents and family cohesion, as well as fewer instances of bullying [22,46]. Shaw et al. found that girls who ate with their family every day showed more resilience to cyberbullying than girls who ate with their families less than weekly [47]. This suggests that family meals offer opportunities to address social and emotional issues, promoting the use of adequate coping strategies, resilience, and family and social support.

Furthermore, for mother–daughter relationships at age 13 years, family meals were found to have a non-linear relationship with early childhood screen time trends. More specifically, the mealtime environment forecasted some benefits. The findings suggest that, for girls, mealtime might be an opportunity to talk and express feelings with mothers. When preschool screen time increased, a pleasant atmosphere in which family members can confide in each other significantly amplified subsequent open communication with mothers about feelings, plans for the future, and daily events. These benefits of increasing screen time trends on mother–daughter relationships could be attributed to the use of screen time as a regulative and relational parenting tool [48]. Specifically, mothers may use screens as a reward or as a facilitator for communication, which is associated with greater screen time [49]. Offering a safe and consistent space for sharing at mealtime may reinforce the foundation of parent and child trust, fostering better communication and positive feelings in children [18]. Although this is mere speculation, all other findings seem to point in this direction.

Surprisingly, increasing screen time trends were associated with fewer instances of peer victimization. For girls, lower family meal environment quality represented a protective factor in this association. Namely, when early childhood screen time increased, girls with low and moderate mealtime environment quality at school entry reported fewer instances of being insulted by peers, excluded from groups, and laughed at by others at age 13 years. This interaction could arise from the way screens are utilized. Early screen time habits often forecast greater digital media use (electronic games, Internet, social media, etc.) in adolescence, particularly for girls, seeing as preferred screens typically shift from television to digital media devices [8,50,51]. Given that forming relationships through screen media allows adolescents to have a platform where they can comfortably connect many people at once, it is plausible that the observed interaction with peer relationships could be explained by the greater number of social relationships, although superficial, that stem from a greater online presence [52,53]. Girls with better social skills acquired through meal sharing could favor forming fewer, but deeper, meaningful connections with individuals, face-to-face.

For boys, the degree to which family meals are perceived as enjoyable did not have a significant interaction with early childhood screen time habits in the associations with later social outcomes. This could be attributed to the gender differences in socialization, placing differential importance on certain experiences, such as family meals, for girls and boys [29,32]. Girls and boys also have preferences in the type of digital media used, which can ultimately have different associations with social development. Boys often prefer digital gaming which parents associate with more negative beliefs, consequently employing parenting strategies based on control rather than on communication [30,51,54]. These negative beliefs are also reflected in our findings where greater increases in preschool screen time forecasted fewer positive relationships with both parents for adolescent boys. 

### Strengths and Limitations

Our findings are limited by several methodological challenges. First, using a population-based longitudinal study for secondary analyses precludes statements of causality, but correlation between life experiences and well-being are still implied. Nevertheless, this research design reflects a natural experiment predating recommendations on screen media restrictions that originate in 2001, reducing social desirability bias [55]. Second, being limited to the data collected as part of the study on child development, we were unable to account for televiewing content or context, as well as life experiences between the ages 6 and 13 years that may have influenced social relationships. Screen time at age 6 years was not as thoroughly measured as at age 2 years. Nevertheless, the television represents the main source of screen time through to age 8 years, even more so at the time of data collection, between 2000 and 2004 [56]. Third, parent-reported family meal environment quality and conflictual parent–child relationships had low internal consistency, evoking the possibility of social desirability bias. Still, we found that screen time habits and family meal environment, as well as the interaction of these two experiences had significant influences on later social relationships in adolescent girls.

Despite these limitations, this study documents how family life experiences can influence different personal characteristics. As a first, we address the sway of early screen time trends on key social relationships past school entry while also examining the role of shared family meals on this association. Moreover, by adopting a person-centered approach, our analyses are more sensitive to continuously measured individual and environmental characteristics that shape the life course. This reduces the weight of alternate explanations to the associations observed in our study. We are suggesting an easy and manageable activity to incorporate into the family life to alter long-term influences of early childhood screen time trends on social relationships. Lastly, girls and boys were treated as separate populations to account for their distinct experiences.

## 5. Conclusions

Shifts to sedentary screen time as a hobby have become a global concern. Enhancing family meal environment represents a simple and cost-efficient activity that can compensate long-term risks of social maladjustment associated with increased screen use. Notably, parents can integrate family meals into the household routine and create an enjoyable, non-rushed, meal sharing ritual by limiting distractions such as screens during meals [57]. Parents and children can use this time to confide in each other, interact with one another about various topics, and promote social skills. Public health initiatives should consider family meals as a complementary intervention strategy to screen use guidelines.

## Figures and Tables

**Table 1:** Descriptive Statistics for Predictor, Moderator, Control, and Outcome Variables.

	*Girls*			*Boys*		
	*M (SD)*	*Categorical Variables (%)*	*Range*	*M (SD)*	*Categorical Variable (%)*	*Range*
*Predictor*						
Screen time (2 years)	1.75 (1.17)		0.00–9.43	1.78 (1.27)		0.00–9.00
Screen time (6 years)	3.00 (1.16)		0.00–8.71	3.09 (1.12)		0.14–7.71
Screen time trend (2 to 6 years)	1.25 (1.41)		−3.43–8.51	1.31 (1.50)		−5.14–7.29
*Moderator (6 years)*						
Family meal environment quality	3.42 (0.32)		1.00–4.00	3.41 (0.33)		1.00–4.00
*Dependent variables (13 years)*						
Positive relationship with mother	7.47 (1.37)		0.00–10.00	7.16 (1.33)		0.00–10.00
Positive relationship with father	6.03 (1.30)		0.00–10.00	5.96 (1.15)		0.00–10.00
Conflictual relationship with mother	2.55 (1.38)		0.00–10.00	2.83 (1.20)		0.00–10.00
Conflictual relationship with father	2.44 (1.20)		0.00–10.00	2.65 (0.99)		0.00–10.00
Victimization	1.35 (0.37)		1.00–5.00	1.47 (0.40)		1.00–5.00
*Pre-existing and concurrent control variables*						
Maternal depressive symptoms (5 months)						
1 = above the median		42.4			47.5	
Parent antisocial antecedents (5 months)						
1 = above the median		47.0			49.6	
Maternal education (5 months)						
1 = did not finish high school		15.4			12.5	
Child BMI (1.5 years)						
1 = above the median		45.2			48.3	
Child temperament problems (1.5 years)						
1 = above the median		52.5			47.1	
Maternal BMI (1.5 years)						
1 = above the median		51.4			51.1	
Family dysfunction (1.5 years)						
1 = above the median		57.0			57.4	
Neurocognitive skills (2 years)						
0 = Score of 0 1 = Score of 1 2 = Score of 2 3 = Score of 3		17.8 54.5 21.9 5.8			20.3 55.0 21.7 2.9	
Family configuration (2 years)						
1 = non-intact		12.1			13.9	
Family income (2 years)						
1 = insufficient revenue		17.1			15.4	
Effective parenting practices (2 years)						
1 = below the median		44.3			44.3	
Screen time (13 years)						
1 = above the recommendations		67.2			70.6	

Notes. M = mean; SD = standard deviation; BMI = Body Mass Index. Analyses corrected for attrition bias. Data were compiled from the final master file of the Québec Longitudinal Study of Child Development (1998–2011), ©Gouvernement du Québec, Institut de la statistique du Québec.

**Table 2:** Unstandardized Regression Coefficients (Standard Error) Reflecting the Adjusted Relationship Between Baseline Child and Family Characteristics Between Ages 5 Months and 13 Years and Screen Time Trend from Ages 2 to 6 Years and Family Meal Environment Quality at Age 6 Years.

	Screen Time Trend (2 to 6 Years)		Family Meal Environment Quality (6 Years)	
Sex	−0.06 (0.08)		0.00 (0.02)	
	***Girls***	***Boys***	***Girls***	***Boys***
Maternal depressive symptoms (5 months)	−0.18 (0.11)	0.01 (0.12)	−0.02 (0.02)	−0.04 (0.02)
Parent antisocial antecedents (5 months)	0.23 (0.11) *	−0.05 (0.11)	−0.03 (0.02)	−0.03 (0.02)
Maternal education (5 months)	−0.37 (0.15) **	−0.26 (0.18)	0.03 (0.03)	0.06 (0.04)
Child BMI (1.5 years)	−0.02 (0.11)	−0.04 (0.11)	0.01 (0.02)	0.00 (0.02)
Child temperament problems (1.5 years)	0.02 (0.11)	0.11 (0.12)	−0.04 (0.02)	−0.09 (0.03) ***
Maternal BMI (1.5 years)	0.17 (0.10)	−0.18 (0.11)	−0.02 (0.02)	0.01 (0.02)
Family dysfunction (1.5 years)	0.16 (0.11)	0.04 (0.12)	−0.21 (0.02) ***	−0.18 (0.02) ***
Neurocognitive skills (2 years)	0.11 (0.07)	−0.01 (0.08)	−0.01 (0.01)	−0.02 (0.02)
Family configuration (2 years)	0.22 (0.18)	−0.23 (0.18)	−0.02 (0.04)	0.03 (0.04)
Family income (2 years)	−0.21 (0.16)	−0.37 (0.17) *	−0.04 (0.03)	−0.08 (0.04) *
Effective parenting practices (2 years)	0.01 (0.11)	0.18 (0.11)	−0.07 (0.02) **	−0.12 (0.02) ***
Concurrent screen time (13 years)	0.19 (0.11)	0.22 (0.13)	−0.06 (0.02) *	−0.03 (0.03)
R^2^	0.037 **	0.034 *	0.171 ***	0.164 ***

Notes. * *p* ≤ 0.05, ** *p* ≤ 0.01, *** *p* ≤ 0.001; two-tailed test. BMI = Body Mass Index. Analyses corrected for attrition bias. Data were compiled from the final master file of the Québec Longitudinal Study of Child Development (1998–2011), ©Gouvernement du Québec, Institut de la statistique du Québec.

**Table 3:** Unstandardized Regression Coefficients (Standard Error) Reflecting the Adjusted Relationship Between Screen Time Trends from Ages 2 to 6 Years and Social Relationship Outcomes at Age 13 Years, Moderated by Family Meal Environment Quality at Age 6 Years for Girls.

	Age 13 Years				
	*Positive Relationship with Mother*	*Positive Relationship with Father*	*Conflictual Relationship with Mother*	*Conflictual Relationship with Father*	*Victimization*
Screen time trend (2 to 6 years) × Family meal environment quality (6 years)	0.25 (0.09) **	0.03 (0.09)	−0.19 (0.10) *	−0.15 (0.09)	0.12 (0.03) ***
Screen time trend (2 to 6 years)	0.05 (0.03)	−0.01 (0.03)	−0.03 (0.04)	−0.07 (0.03) *	−0.02 (0.01) *
Family meal environment quality (6 years)	1.18 (0.16) ***	0.73 (0.15) ***	−1.03 (0.16) ***	−0.71 (0.14) ***	−0.10 (0.04) *
Maternal depressive symptoms (5 months)	−0.03 (0.10)	−0.29 (0.10) **	0.08 (0.10)	−0.15 (0.09)	−0.01 (0.03)
Parent antisocial antecedents (5 months)	−0.11 (0.09)	−0.14 (0.09)	0.46 (0.10) ***	0.36 (0.09) ***	0.06 (0.03) *
Maternal education (5 months)	−0.13 (0.14)	0.21 (0.13)	0.23 (0.14)	0.14 (0.12)	0.07 (0.04) *
Child BMI (1.5 years)	−0.24 (0.09) **	−0.53 (0.09) ***	0.23 (0.10) *	0.15 (0.09)	0.05 (0.03)
Child temperament problems (1.5 years)	−0.10 (0.10)	−0.04 (0.09)	0.01 (0.10)	0.24 (0.09) **	−0.01 (0.03)
Maternal BMI (1.5 years)	−0.13 (0.09)	−0.08 (0.09)	0.08 (0.10)	0.08 (0.08)	0.05 (0.03) *
Family dysfunction (1.5 years)	−0.21 (0.10) *	−0.30 (0.10) **	−0.09 (0.11)	0.08 (0.09)	−0.01 (0.03)
Neurocognitive skills (2 years)	0.09 (0.06)	0.01 (0.06)	−0.16 (0.06) **	−0.08 (0.05)	−0.02 (0.02)
Family configuration (2 years)	−0.49 (0.16) **	0.13 (0.15)	0.63 (0.16) ***	0.50 (0.14) ***	0.07 (0.04)
Family income (2 years)	0.18 (0.14)	0.27 (0.14) *	0.07 (0.14)	0.00 (0.13)	0.13 (0.04) ***
Effective parenting practices (2 years)	−0.34 (0.10) ***	0.07 (0.09)	−0.02 (0.10)	0.04 (0.09)	0.00 (0.03)
Concurrent screen time (13 years)	−0.22 (0.10) *	−0.30 (0.10) **	0.11 (0.10)	0.09 (0.09)	0.04 (0.03)
R^2^	0.184 ***	0.160 ***	0.151 ***	0.125 ***	0.118 ***

Notes. * *p* ≤ 0.05, ** *p* ≤ 0.01, *** *p* ≤ 0.001; two-tailed test. BMI = Body Mass Index. Analyses corrected for attrition bias. Data were compiled from the final master file of the Québec Longitudinal Study of Child Development (1998–2011), ©Gouvernement du Québec, Institut de la statistique du Québec.

**Table 4:** Unstandardized Regression Coefficients (Standard Error) Reflecting the Adjusted Relationship Between Screen Time Trends from Ages 2 to 6 Years and Social Relationship Outcomes at Age 13 Years, Moderated by Family Meal Environment Quality at Age 6 Years for Boys.

	Age 13 Years				
	*Positive Relationship with Mother*	*Positive Relationship with Father*	*Conflictual Relationship with Mother*	*Conflictual Relationship with Father*	*Victimization*
Screen time trend (2 to 6 years) × Family meal environment quality (6 years)	−0.02 (0.09)	0.07 (0.08)	0.03 (0.09)	−0.05 (0.07)	0.02 (0.03)
Screen time trend (2 to 6 years)	−0.08 (0.03) **	−0.10 (0.03) ***	−0.02 (0.03)	−0.02 (0.03)	−0.02 (0.01) *
Family meal environment quality (6 years)	1.10 (0.15) ***	0.73 (0.13) ***	−0.58 (0.14) ***	−0.40 (0.12) ***	−0.16 (0.05) ***
Maternal depressive symptoms (5 months)	0.00 (0.10)	0.29 (0.08) ***	0.06 (0.09)	−0.12 (0.08)	0.07 (0.03) *
Parent antisocial antecedents (5 months)	−0.19 (0.09) *	0.03 (0.08)	0.33 (0.09) ***	0.06 (0.07)	0.06 (0.03) *
Maternal education (5 months)	−0.24 (0.15)	0.20 (0.13)	0.09 (0.14)	0.41 (0.12) ***	0.09 (0.05) *
Child BMI (1.5 years)	0.00 (0.09)	−0.01 (0.08)	−0.10 (0.09)	−0.30 (0.07) ***	0.02 (0.03)
Child temperament problems (1.5 years)	−0.30 (0.10) **	−0.19 (0.09) *	0.15 (0.10)	0.10 (0.08)	−0.06 (0.03) *
Maternal BMI (1.5 years)	−0.25 (0.09) **	−0.36 (0.08) ***	0.26 (0.09) **	−0.11 (0.07)	0.01 (0.03)
Family dysfunction (1.5 years)	−0.13 (0.10)	−0.13 (0.09)	−0.10 (0.10)	−0.15 (0.08)	−0.03 (0.03)
Neurocognitive skills (2 years)	0.00 (0.07)	0.09 (0.05)	−0.15 (0.06) **	0.10 (0.05) *	−0.04 (0.02) *
Family configuration (2 years)	0.06 (0.15)	−0.87 (0.13) ***	0.19 (0.14)	0.26 (0.12) *	−0.13 (0.05) **
Family income (2 years)	0.16 (0.15)	−0.16 (0.12)	0.14 (0.14)	−0.47 (0.11) ***	0.07 (0.05)
Effective parenting practices (2 years)	−0.07 (0.10)	−0.08 (0.08)	0.22 (0.09) **	−0.01 (0.08)	−0.03 (0.03)
Concurrent screen time (13 years)	−0.17 (0.11)	−0.07 (0.09)	−0.04 (0.10)	−0.11 (0.08)	−0.14 (0.03) ***
R^2^	0.149 ***	0.191 ***	0.097 ***	0.088 ***	0.083 ***

Notes. * *p* ≤ 0.05, ** *p* ≤ 0.01, *** *p* ≤ 0.001; two-tailed test. BMI = Body Mass Index. Analyses corrected for attrition bias. Data were compiled from the final master file of the Québec Longitudinal Study of Child Development (1998–2011), ©Gouvernement du Québec, Institut de la statistique du Québec.

## Data Availability

The data presented in this study are available on request from the Institut de la Statistique du Québec (ISQ). The data are not publicly available due to permission of the ISQ.

## Appendix A. Missing Data Analysis

Comparative analyses using chi-squared tests highlighted the differences between the participants with complete and incomplete data at age 13 years on individual and demographic measures. Compared with participants with incomplete data, those with complete data on social relationship outcomes at age 13 years had mothers with fewer depressive symptoms, *X*^2^ (1, *N* = 1472) = 30.300, *p* < 0.001, 95% CI [1.463, 2.234] and that were more educated *X*^2^ (1, *N* = 1476) = 13.185, *p* < 0.001, 95% CI [1.304, 2.461]. They also had parents who reported less antisocial behaviors, *X*^2^ (1, *N* = 1453) = 4.762, *p* < 0.05, 95% CI [1.024, 1.555]. Comparisons also revealed that cases with complete data belonged to fewer dysfunctional families, *X*^2^ (1, *N* = 1412) = 10.737, *p* < 0.001, 95% CI [1.153, 1.764], more intact families, *X*^2^ (1, *N* = 1477) = 771.692, *p* < 0.001, 95% CI [3.523, 8.287], with sufficient family income *X*^2^ (1, *N* = 1456) = 41.194, *p* < 0.001, 95% CI [2.027, 3.875]. Girls had more complete data than boys, *X*^2^ (1, *N* = 1455) = 12.581, *p* < 0.001, 95% CI [0.555, 0.844].

Girls with mothers experiencing more depressive symptoms and with mothers who had not obtained a high school diploma were less likely to have complete data, *X*^2^ (1, *N* = 739) = 14.752, *p* < 0.001, 95% CI [1.326, 2.401] and *X*^2^ (1, *N* = 740) = 11.826, *p* < 0.001, 95% CI [1.363, 3.189], respectively. Girls belonging to families with higher levels of dysfunction, *X*^2^ (1, *N* = 711) = 6.773, *p* < 0.01, 95% CI [1.102, 1.998], non-intact families, *X*^2^ (1, *N* = 741) = 41.783, *p* < 0.001, 95% CI [3.317, 11.178], and households with insufficient income, *X*^2^ (1, *N* = 731) = 22.296, *p* < 0.001, 95% CI [1.784, 4.214] had more incomplete data.

Boys with complete data had less depressed mothers, *X*^2^ (1, *N* = 712) = 13.654, *p* < 0.001, 95% CI [1.312, 2.435], parents with less antisocial antecedents, *X*^2^ (1, *N* = 703) = 5.145, *p* < 0.05, 95% CI [1.049, 1.938], and mothers with a lower BMI, *X*^2^ (1, *N* = 705) = 10.673, *p* < 0.001, 95% CI [0.440, 0.815]. These boys were also more likely to come from non-intact families, *X*^2^ (1, *N* = 714) = 28.326, *p* < 0.001, 95% CI [2.508, 8.410] and from a household with sufficient income, *X*^2^ (1, *N* = 704) = 18.039, *p* < 0.001, 95% CI [1.737, 4.752].

## Appendix B. Direct Association Results

Table 3 and Table 4 document the unstandardized regression coefficients with standard errors reflecting the adjusted relationship between screen time trends from ages 2 to 6 years and social relationship outcomes at age 13 years, moderated by family meal environment quality at age 6 years for girls and for boys, respectively. Higher increases in daily screen time predicted less conflictual father–daughter relationships (β = −0.07, *p* ≤ 0.05, 95% CI, −0.12 to 0.00), more positive mother–son (β = −0.09, *p* ≤ 0.01, 95% CI, −0.14 to −0.02) and father–son relationships (β = −0.13, *p* ≤ 0.001, 95% CI, −0.15 to −0.05), and less peer victimization for both girls and boys (β_girls_ = −0.08, *p* ≤ 0.05, 95% CI, −0.04 to 0.00; β_boys_ = −0.08, *p* ≤ 0.05, 95% CI, −0.04 to 0.00). Higher family meal environment quality was associated with more positive relationships with mothers (β_girls_ = 0.28, *p* ≤ 0.001, 95% CI, 0.87 to 1.49; β_boys_ = 0.28, *p* ≤ 0.001, 95% CI, 0.79 to 1.40) and fathers (β_girls_ = 0.18, *p* ≤ 0.001, 95% CI, 0.43 to 1.03; β_boys_ = 0.21, *p* ≤ 0.001, 95% CI, 0.47 to 0.98), less conflictual relationships with mothers (β_girls_ = −0.24, *p* ≤ 0.001, 95% CI, −1.35 to −0.71; β_boys_ = −0.16, *p* ≤ 0.001, 95% CI, −0.86 to −0.30) and fathers (β_girls_ = −0.19, *p* ≤ 0.001, 95% CI, −0.99 to −0.42; β_boys_ = −0.14, *p* ≤ 0.001, 95% CI, −0.63 to −0.16), and less victimization by classmates (β_girls_ = −0.09, *p* ≤ 0.05, 95% CI, −0.19 to −0.02; β_boys_ = −0.13, *p* ≤ 0.001, 95% CI, −0.26 to −0.07). These associations are significant above and beyond the influence of many confound control variables.
